# Late-onset diaphragmatic hernia after percutaneous radiofrequency ablation of hepatocellular carcinoma: a case study

**DOI:** 10.1186/s40792-016-0148-3

**Published:** 2016-03-14

**Authors:** Tomoyuki Abe, Hironobu Amano, Hitomi Takechi, Nobuaki Fujikuni, Tatsunari Sasada, Makoto Yoshida, Minoru Yamaki, Masahiro Nakahara, Toshio Noriyuki

**Affiliations:** Department of Surgery, Onomichi General Hospital, Onomichi, Hiroshima Japan; Department of Gastroenterological and Transplant Surgery, Applied Life Sciences, Institute of Biomedical and Health Sciences, Hiroshima University, Hiroshima, Japan

**Keywords:** Diaphragmatic hernia, Hepatocellular carcinoma, Radiofrequency ablation

## Abstract

Percutaneous radiofrequency ablation (RFA) is widely used as an effective treatment of liver tumors. Several reported complications associated with RFA are due to thermal damage of neighboring organs. The present report presents a case of diaphragmatic hernia associated with RFA and hepatocellular carcinoma (HCC). A 72-year-old woman with S5 and S8 HCCs was treated repeatedly with RFA and transcatheter arterial chemoembolization for 3 years. After the third course of RFA to target the recurring S5 HCC, acute abdominal pain and dyspnea suddenly occurred. Contrast-enhanced computed tomography revealed intrusion of the transverse colon through the right diaphragmatic hernia. In addition, the colon was dilated and showed changes suggestive of ischemic conditions. An emergency surgery was performed to close the hernia by using non-absorbable sutures to preserve the colon. The patient was discharged without any complications 13 days after the surgery. The first-line treatment of this disease involves surgical intervention. Diaphragmatic hernia is a rare complication of RFA. The present case suggests that patients who undergo several rounds of RFA require surveillance for diaphragmatic hernias.

## Background

The incidence of hepatocellular carcinoma (HCC) has been increasing worldwide, making HCC one of the most common malignant tumors [1]. Liver resection provides enhanced long-term survival, with a 5-year overall survival rate of around 60 % [2, 3]. These results are reflected by developments in the surgical techniques and perioperative management approaches for HCC. Liver resection is the first-line intervention for patients with preserved liver function and fewer than three liver tumors. Alternative treatments include radiofrequency ablation (RFA) and transarterial chemoembolization (TACE). RFA is widely accepted as a minimally invasive treatment of HCC, with low risk of complications, especially when compared with liver resection (LR). However, the efficacy of RFA is inferior to that of LR [4, 5]. Reported complications of RFA include bleeding, abscess formation, biliary fistula, pneumothorax, hepatic insufficiency, and, more infrequently, diaphragmatic hernia [6–8]. Herein, we present a rare case of delayed diaphragmatic hernia due to frequently performed RFA for HCC treatment.

## Case presentation

**Fig. 1:**
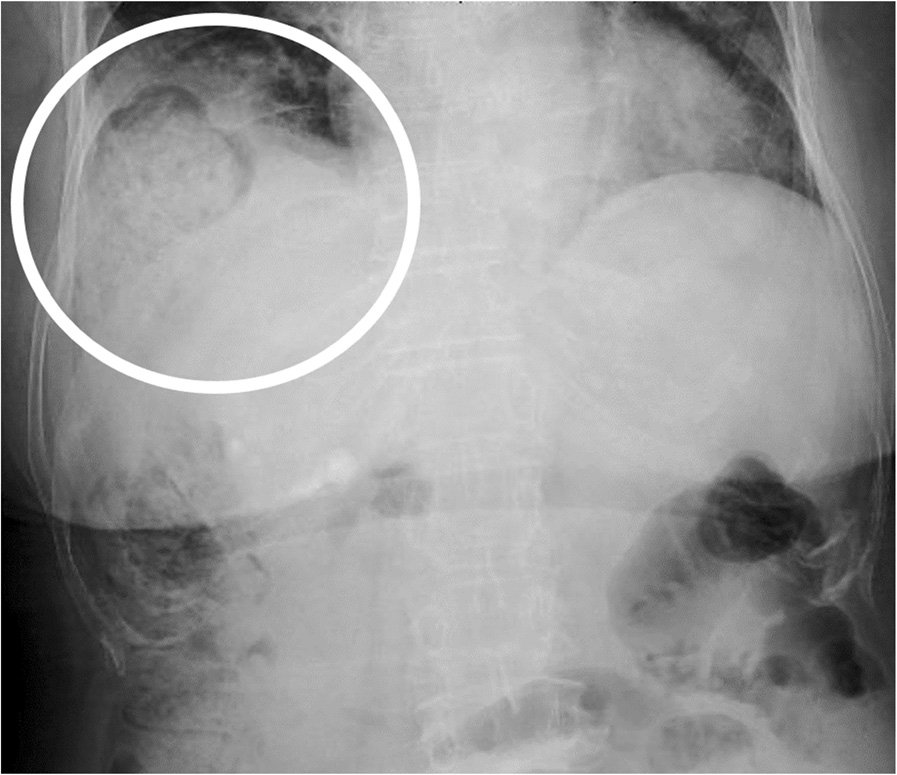
Abdominal radiograph obtained at symptom onset, showing the deviation of the colon across the right diaphragm (*white circle*)

**Fig. 2:**
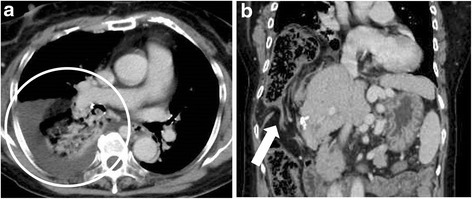
**a** The transverse colon is inserted in the right pleural cavity through the diaphragmatic hernia. The hernia is 10 cm in diameter. Pleural effusion is detected in the right chest cavity (*white circle*). **b** The massive transverse colon is intruded into the right chest cavity. Severe stricture can be observed (*white arrow*)

**Fig. 3:**
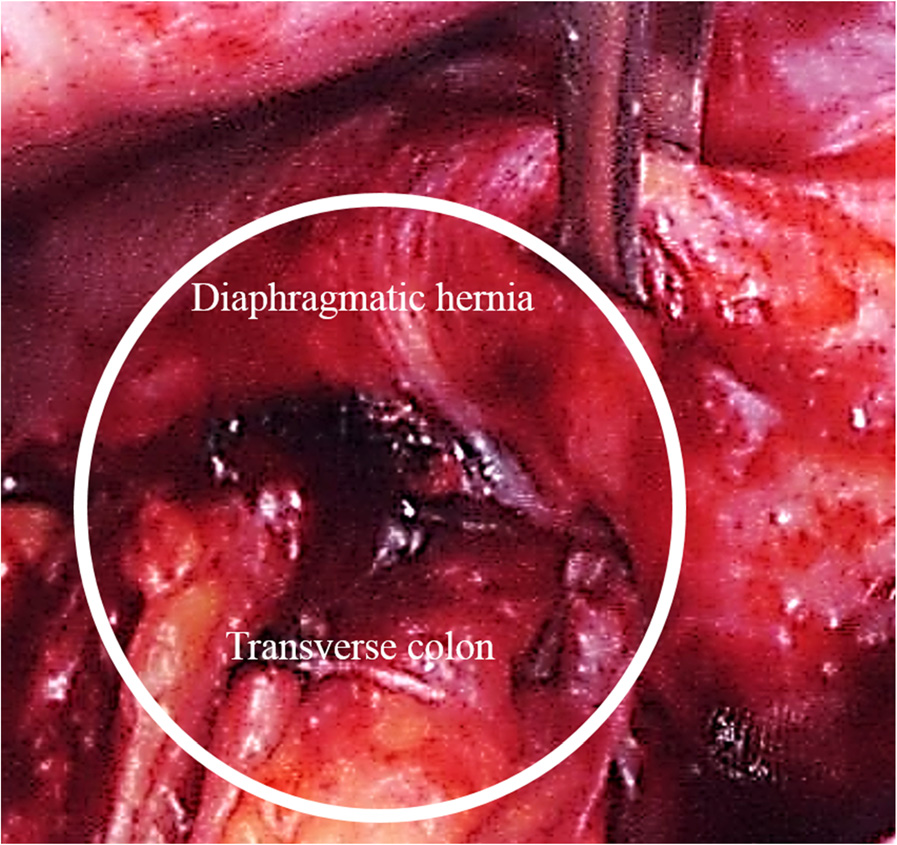
The transverse colon is directly intruded into the right thoracic cavity through the diaphragmatic hernia (*white circle*)

A 72-year-old woman was admitted to our hospital because of acute pain in the right hypochondrium. In 2011, she was diagnosed with hepatitis C virus-related liver cirrhosis (Child-Pugh grade B) and HCC. RFA and TACE were first introduced for S5 HCC. After the first treatment, the HCC did not recur; however, S5 HCC recurrence was detected on dynamic computed tomography (CT) in September 2013. Follow-up CT was performed every 3 months after the RFA. After several courses of RFA therapy targeting the recurring HCC, a sudden, strong right upper abdominal pain and dyspnea occurred in 2015. The last RFA therapy session was performed 15 months before the symptom onset. Abdominal radiography revealed a massive abnormal free air on the right side under the diaphragm (Fig. 1). Hence, the patient was transferred to our hospital under the suspicion of acute abdomen. The vital signs were not remarkable as follows: blood pressure, 136/90 mmHg; pulse rate, 107/min; and SpO_2_, 96 % (room air). In a physical examination, acute pain was found in the right hypochondrium. Laboratory findings did not demonstrate any abnormalities, including white blood cell count or C-reactive protein level. Chest radiograms showed lower right pulmonary opacity and left mediastinal shift. Contrast-enhanced CT revealed that the transverse colon was intruded through a diaphragmatic hernia and the colon was well enhanced (Fig. 2a, b). As the diagnosis included a right diaphragmatic hernia with suspicion of a strained colon, an emergency surgery was performed. The surgery revealed a transverse colon inserted in the right pleural cavity via the diaphragmatic hernia, which was 10 cm in diameter (Fig. 3). The location of the RFA-treated tumor matched the site of the diaphragmatic hernia. The intruded transverse colon did not show necrosis. Thus, after dissolving the hernial intrusion, the hernia was simply closed by using non-absorbable sutures. The overall surgery time was 85 min, and the amount of bleeding was 650 ml. After the surgery, the patient was discharged without any complications.

Diaphragmatic hernia is a rare late-onset complication associated with RFA. Repetitive RFA for HCC makes the diaphragm fragile due to thermal damage [6, 9]. The present case required an emergency surgery because the transverse colon was intubated through the diaphragmatic hernia. After three courses of RFA, contrast-enhanced CT revealed the sudden emergence of a right diaphragmatic hernia. Shibuya et al. suggested the rarity of late-onset diaphragmatic hernia, highlighting that only six cases of RFA-related iatrogenic diaphragmatic hernia have been reported [10]. Recent reports have shown that laparoscopic methods of diaphragmatic repair have been effective in elective settings [7]. The surgical approach could depend on the timing of the surgery and the association with other organ intrusions of the hernia, making it difficult to obtain a working space for laparoscopic repair. To prevent additional damage due to hernial intrusion, this disease should be treated as soon as diagnosis is confirmed.

In general, according to liver function and tumor stage, percutaneous treatment is selected for local liver tumors; however, its efficacy could not match to that of other methods such as LR and liver transplantation [5, 11]. Percutaneous RFA treatment was established as a promising therapy to maintain local liver malignancy; however, both acute and late-onset complications have been reported [12, 13]. In a study conducted in Italy, 2320 patients with 3554 lesions were enrolled, of whom 1610 patients had HCC with abnormal liver disease. Of the 1610 patients, six died owing to complications, including intestinal perforation, septic shock, tumor rupture, liver failure, and other unknown causes [9]. Major complications were intraperitoneal hemorrhage and intrahepatic abscess. RFA complications were subdivided into four categories as follows: thermal damage from heating and mechanical, septic, and other unexplained causes [6]. Regarding diaphragmatic hernia, only six cases associated with RFA have been reported (Table 1) [7, 10, 14–17]. Of these cases, one was treated conservatively and the rest required surgical intervention. Preventing this complication is impossible, but early diagnosis is possible by meticulous follow-up. Special attention should be paid in the occurrence of diaphragmatic hernia in patients who frequently receive RFA therapy in the right lobe, which is widely covered by the diaphragm.

**Table 1:** Reported cases of diaphragmatic hernia occurring after radiofrequency ablation

No.	Author	Year	Age (years)	Sex	Location of HCC	Time from latest RFA (month)	Treatment	Complications	Outcomes
1	Koda et al. [14]	2002	61	F	sIV	13	OS	HCC rupture	Dead
2	Shibuya et al. [10]	2006	72	M	Border of sIV and sVIII	18	OS	None	Alive
3	di Francesco et al. [16]	2008	49	M	Dome of the right lobe	15	OS	None	Alive
4	Yamagami et al. [15]	2010	71	F	sVII	9	None	None	Alive
5	Singh et al. [17]	2011	46	F	Border of sV and sVII	11	LS	None	Alive
6	Nomura et al. [7]	2014	62	M	sVIII	96	LS	None	Alive
7	Our case	2016	72	F	sVIII	15	OS	None	Alive

*F* female, *LS* laparoscopic surgery, *M* male, *OS* open surgery

The clinical characteristics of diaphragmatic hernia include right upper quadrant abdominal acute pain with dyspnea. Chest radiographic findings are sometimes misdiagnosed as gastrointestinal perforation because the colon gas in the chest wall may be disguised as abdominal free air. Contrast-enhanced CT plays an important role in the diagnosis and in determining if the intruded organs are necrotic. As diaphragmatic hernia requires surgical repair, recent reports demonstrated that laparoscopic simple closure is effective [7]. The laparoscopic approach for an acute abdomen is less invasive and promotes a faster recovery in patients with stable vital signs and no previous medical history of surgery [18]. Timely and accurate diagnosis of this disease is important because these patients have severe liver dysfunction; therefore, colon necrosis leads to poor prognosis even after curative treatment. Once diagnosed, surgical repair is required to prevent intestinal intrusion.

## Conclusions

In conclusion, the present case suggests that frequent RFA therapy may render the right diaphragm fragile due to the frequent thermal damage. Special attention to diagnose delayed complications of RFA, such as diaphragmatic hernia, is warranted in such cases.

## Consent

Written informed consent was obtained from the patient for publication of this case report and accompanying images. A copy of the written consent is available for review by the editor-in-chief of the Journal.

## Abbreviations

CT: computed tomography
HCC: hepatocellular carcinoma
LR: liver resection
RFA: radiofrequency ablation
TACE: transarterial chemoembolization

## References

[1] Forner A, Llovet JM, Bruix J (2012). Hepatocellular carcinoma. Lancet.

[2] Fan ST, Mau Lo C, Poon RT, Yeung C, Leung Liu C, Yuen WK (2011). Continuous improvement of survival outcomes of resection of hepatocellular carcinoma: a 20-year experience. Ann Surg.

[3] Kuroda S, Tashiro H, Kobayashi T, Oshita A, Amano H, Ohdan H (2012). No impact of perioperative blood transfusion on recurrence of hepatocellular carcinoma after hepatectomy. World J Surg.

[4] Imai K, Beppu T, Chikamoto A, Doi K, Okabe H, Hayashi H (2013). Comparison between hepatic resection and radiofrequency ablation as first-line treatment for solitary small-sized hepatocellular carcinoma of 3 cm or less. Hepatol Res.

[5] Huang J, Yan L, Cheng Z, Wu H, Du L, Wang J (2010). A randomized trial comparing radiofrequency ablation and surgical resection for HCC conforming to the Milan criteria. Ann Surg.

[6] Rhim H, Yoon KH, Lee JM, Cho Y, Cho JS, Kim SH (2003). Major complications after radio-frequency thermal ablation of hepatic tumors: spectrum of imaging findings. Radiographics.

[7] Nomura R, Tokumura H, Furihata M (2014). Laparoscopic repair of a diaphragmatic hernia associated with radiofrequency ablation for hepatocellular carcinoma: lessons from a case and the review of the literature. Int Surg.

[8] Wood TF, Rose DM, Chung M, Allegra DP, Foshag LJ, Bilchik AJ (2000). Radiofrequency ablation of 231 unresectable hepatic tumors: indications, limitations, and complications. Ann Surg Oncol.

[9] Livraghi T, Solbiati L, Meloni MF, Gazelle GS, Halpern EF, Goldberg SN (2003). Treatment of focal liver tumors with percutaneous radio-frequency ablation: complications encountered in a multicenter study. Radiology.

[10] Shibuya A, Nakazawa T, Saigenji K, Furuta K, Matsunaga K (2006). Diaphragmatic hernia after radiofrequency ablation therapy for hepatocellular carcinoma. AJR Am J Roentgenol.

[11] Hasegawa K, Makuuchi M, Takayama T, Kokudo N, Arii S, Okazaki M (2008). Surgical resection vs. percutaneous ablation for hepatocellular carcinoma: a preliminary report of the Japanese nationwide survey. J Hepatol.

[12] Kim JS, Kim HS, Myung DS, Lee GH, Park KJ, Cho SB (2013). A case of diaphragmatic hernia induced by radiofrequency ablation for hepatocellular carcinoma. Korean J Gastroenterol.

[13] Francica G (2001). Complications of radio-frequency thermal ablation. Radiology.

[14] Koda M, Ueki M, Maeda N, Murawaki Y (2003). Diaphragmatic perforation and hernia after hepatic radiofrequency ablation. AJR Am J Roentgenol.

[15] Yamagami T, Yoshimatsu R, Matsushima S, Tanaka O, Miura H, Nishimura T (2011). Diaphragmatic hernia after radiofrequency ablation for hepatocellular carcinoma. Cardiovasc Intervent Radiol.

[16] di Francesco F, di Sandro S, Doria C, Ramirez C, Iaria M, Navarro V (2008). Diaphragmatic hernia occurring 15 months after percutaneous radiofrequency ablation of a hepatocellular cancer. Am Surg.

[17] Singh M, Singh G, Pandey A, Cha CH, Kulkarni S (2011). Laparoscopic repair of iatrogenic diaphragmatic hernia following radiofrequency ablation for hepatocellular carcinoma. Hepatol Res.

[18] Abe T, Kajiyama K, Harimoto N, Gion T, Nagaie T (2012). Laparoscopic omentectomy for preoperative diagnosis of torsion of the greater omentum. Int J Surg Case Rep.

